# Prediction of post-partum depression and anxiety based on clinical interviews and symptom self-reports of depression and anxiety during pregnancy

**DOI:** 10.1192/j.eurpsy.2022.688

**Published:** 2022-09-01

**Authors:** E. Wilkie, V. Gillet, A. Talati, J. Posner, L. Takser

**Affiliations:** 1 Sherbrooke University, Pediatrics, Sherbrooke, Canada; 2 Columbia University Medical Center and New York Psychiatric Institute, Psychiatry, New York, United States of America; 3 Duke University, Department Of Psychiatry, Durham, United States of America

**Keywords:** Depression, Psychometric measures, Anxiety, Postpartum

## Abstract

**Introduction:**

The tools used to evaluate mental health during pregnancy matter. Their efficacy in identifying symptom severity enables better predictions of postpartum mental health. The Mother & Youth: Research on Neurodevelopment & behaviour (MYRNA) cohort is an NIH funded longitudinal cohort from Sherbrooke, Canada studying the effects of pregnant women’s mental health.

**Objectives:**

We examine which mental health tools will better gauge depression and anxiety during pregnancy based on predicting postpartum outcomes. Our hypothesis is that an approach combining a clinical interview with self-report questionnaires may predict mental health in postpartum women.

**Methods:**

Participants’ mental health is evaluated by the SCID-5-RV, a lifetime interview administered at 30 weeks and monthly questionnaires including PHQ-9 and GAD-7. Participants are in the depression/anxiety group if they either pass all the criteria in the SCID during pregnancy or have an average PHQ-9 or GAD-7 score greater than 7. The Edinburgh Postnatal Depression Scale (EPDS) and the Perceived Stress Scale (PSS) are the outcome variables.

**Results:**

PHQ-9 was correlated with EPDS, *r*(220)= .38, *p*< .01, and GAD-7 was correlated with PSS, *r*(213)= .56, p< .01. SCID results only had a significant effect on PSS, *F*(3,220)= 3.77, *p*= .01 and not with EPDS, *F*(3,219)= 1.08, *p*= .36. When the self-report measures and interview were combined significant effects were seen for both the EPDS, *F*(1,222)= 18.71, *p*< .01 and the PSS, *F*(1,223)= 34.94, *p*<.01.

**Conclusions:**

Preliminary results show significant associations between measures administered during pregnancy and postpartum measures. Prediction models based on classification will be analyzed once more data is collected.

**Disclosure:**

No significant relationships.

